# Septic Arthritis of the Knee in Children

**DOI:** 10.7759/cureus.45659

**Published:** 2023-09-21

**Authors:** Hussam Darraj, Khalid M Hakami, Basem Zogel, Rawan Maghrabi, Zenat Khired

**Affiliations:** 1 Surgery, Jazan University, Jazan, SAU; 2 Medicine, Jazan University, Jazan, SAU; 3 Medicine and Surgery, Jazan University, Jazan, SAU

**Keywords:** joint damage, children, inflammation, knee, septic arthritis

## Abstract

Septic arthritis of the knee is the most common form of septic arthritis in children and can lead to irreversible damage to the joint. *Staphylococcus* *aureus* is the primary pathogen associated with septic arthritis, although other causative pathogens may be isolate in children with specific risk factors. The diagnosis of knee septic arthritis is based on comprehensive evaluation, including the patient's medical history, physical examination, blood tests, and arthrocentesis. Empirical treatment typically involves anti-staphylococcal penicillin or a first-generation cephalosporin, although modifications may be made based on local resistance patterns and clinical culture data. Surgical debridement, either through open surgery or arthroscopy, involving extensive debridement of the joint, is effective in eliminating the infection. In most cases, additional surgical intervention is not necessary.

## Introduction and background

Septic arthritis (SA) is characterized by joint infection caused by bacteria, viruses, or fungi, with bacteria being the most common culprits [1, 2]. The incidence of SA in the pediatric population is estimated to range from 4 to 37 cases per 100,000 individuals. Significant variations exist between developed and developing regions, as well as among different age groups [2, 3]. Children under the age of two are particularly susceptible to this condition [4]. The most frequently affected joints in the body are the large joints of the lower limb, including the hip, knee, and ankle joints [5].

Historically, septic arthritis has been associated with poor prognosis and high mortality rates [6,7]. However, advancements in diagnostic and treatment approaches have resulted in reduced morbidity and mortality rates associated with this condition [6, 7]. Septic arthritis of the knee is a debilitating joint disease caused by infection within the joint space, typically accompanied by severe symptoms such as pain and limited range of motion. Timely treatment is crucial to prevent permanent joint damage, which can lead to chronic deformity, mechanical arthritis, and even death [8]. The synovium is primarily involved in this condition, affecting all structures within the joint boundaries [9].

Limited international studies have investigated the risk factors for long-term complications in children with SA [10-12]. A systematic review conducted in 2009 highlighted that young age and delayed treatment are unfavorable prognostic factors in children with SA [13].The purpose of this study (review) is to provide a comprehensive analysis of septic arthritis of the knee in children. By examining the current literature, clinical studies, and available evidence, this review aims to summarize the key aspects of this condition, including its epidemiology, risk factors, clinical presentation, diagnostic approaches, treatment options, and outcomes. Through a systematic analysis of the existing knowledge, this study (review) aims to enhance our understanding of septic arthritis of the knee in children, identify gaps in research, and provide valuable insights for clinicians, researchers, and healthcare professionals involved in the management of this challenging condition.

## Review

Epidemiology

Septic arthritis of the knee is a severe joint infection that, if not promptly treated, can result in permanent damage and disability [14]. To guide research, treatment, and prevention strategies, understanding the incidence and prevalence of this condition is crucial [15]. However, accurately determining these epidemiological measures for septic arthritis presents unique challenges [16].

The incidence of knee septic arthritis in children has been estimated through large cohort studies and reviews of hospital records [17, 18]. Reported incidence rates vary widely, ranging from 2 to 11 cases per 100,000 children per year [19, 20]. Several factors contribute to this variation. Rates are highest among young children (<5 years) and adolescents (8-18 years) [20]. Changes over time can occur due to diagnostic and epidemiological factors [14, 17]. Additionally, differences in case definitions and diagnostic criteria between studies [19, 21], as well as variability in healthcare access and treatment protocols across locations, contribute to the variability in reported incidence rates [22, 17].

A meta-analysis found an overall incidence rate of 4.6 cases per 100,000 person-years for pediatric septic arthritis [23]. However, these estimates likely underestimate the true incidence, particularly in younger children, due to underdiagnosis [24]. Few studies have determined the point prevalence of knee septic arthritis in children, with estimates ranging from 0.01% to 0.08% in school-aged children [19]. However, the true prevalence is likely higher due to factors such as under-recognition and delayed diagnosis, especially in young children, as many cases are diagnosed after the infection has been present for days to weeks [20]. Additionally, subclinical and milder forms may go undetected, and some cases resolve spontaneously without medical intervention [25]. Transient bacteremia and synovial inflammation that does not progress to overt septic arthritis also contribute to the underestimated prevalence [26]. The limitations of retrospective study design with small sample sizes further impact accurate prevalence estimation [27]. Improved awareness, diagnostic testing, and monitoring could help identify more cases and provide more accurate prevalence estimates [21]. However, large prospective studies focusing on high-risk groups are needed to determine the true population prevalence [20].

Pathophysiology

Bacterial Entry and Infection

Bacteria typically enter the joint space through hematogenous spread from a distant site of infection or direct inoculation resulting from trauma or injections [22, 23]. Once inside the joint, bacteria adhere to extracellular matrix proteins and initiate replication [28]. This triggers the activation of the host immune response, leading to the release of pro-inflammatory cytokines such as TNF-α, IL-1β, and IL-6 [29]. Consequently, there is a significant influx of neutrophils into the synovial fluid, causing pronounced synovitis and pain [28, 30].

Effects on Cartilage and Bone

The inflammatory mediators released during septic arthritis contribute to the degradation of cartilage matrix proteins, such as collagen and proteoglycans, through proteolysis [31]. This can result in the loss of joint cartilage and permanent damage, particularly when not promptly treated [32]. Furthermore, the bone around the joint is also affected. Inflammatory cytokines stimulate osteoclastogenesis, leading to bone resorption and juxta-articular osteopenia [33, 34]. Severe and prolonged infection can also cause osteonecrosis and avascular necrosis due to impaired blood supply [35].

Synovial Fluid Changes

Septic arthritis is characterized by specific changes in the synovial fluid: an increased white blood cell count, particularly elevated levels of neutrophils (>50,000 cells/mm3) [28]. Inflammatory markers such as ESR, CRP, and synovial fluid lactate are also elevated [36]. Additionally, bacteria can be identified through staining and culture of the synovial fluid [20]. While gram-positive cocci like *Staphylococcus aureus* are the most common causative organisms, other pathogens can also lead to septic arthritis [37].

Diagnosis 

The diagnostic criteria for pediatric knee septic arthritis encompass several important factors. Clinical features often include the sudden onset of acute knee pain, swelling, warmth, and limited range of motion. Fever is also present in approximately 50-80% of cases. Laboratory findings typically show an elevated white blood cell count and increased levels of erythrocyte sedimentation rate or C-reactive protein. Synovial fluid analysis may reveal a white blood cell count exceeding 50,000 cells/mm3 with more than 90% neutrophils, and bacteria may be isolated through culture. Imaging findings can indicate periarticular bone changes, such as osteopenia, erosions, or soft tissue swelling, on radiographs, and joint effusion may be observed on ultrasound or MRI. The response to antibiotic treatment, characterized by clinical and laboratory improvement, is also considered in the diagnostic criteria [20, 36].

Key tools for diagnosing pediatric knee septic arthritis include synovial fluid analysis, imaging studies, biomarkers, and synovial biopsy. Synovial fluid analysis, which involves cell count and differentials, gram stain, and culture, is particularly valuable. Fluid lactate levels may also assist in differentiating this condition from other causes. Imaging studies, such as ultrasound, CT, and MRI, provide valuable information about the extent of joint involvement and can help identify complications like osteomyelitis. Ultrasound is especially useful for detecting joint effusion, guiding aspiration, and assessing treatment response, while MRI can provide detailed information about soft tissue involvement [28, 38-42]. Biomarkers, specifically elevated inflammatory markers like C-reactive protein (CRP) and erythrocyte sedimentation rate (ESR), are often suggestive but not definitive. Synovial fluid analysis remains the gold standard for diagnosis, with the presence of purulent fluid, an elevated leukocyte count, and positive cultures. Blood cultures can also aid in identifying the causative organism. Synovial biopsy is reserved for refractory cases to detect atypical organisms and rule out autoimmune conditions [28, 38-42].

Treatment

Timely and appropriate treatment is crucial for children with septic arthritis of the knee to prevent permanent joint damage and disability. The main treatment goals are (1) to control the infection; (2) to relieve joint inflammation and swelling; and (3) to preserve joint function and promote healing [20]. These objectives are achieved through a combination of antibiotic therapy, surgical drainage, and joint immobilization.

Antibiotic Therapy

Immediate initiation of empiric antibiotic therapy is crucial once synovial fluid and blood cultures have been obtained [38, 43]. Commonly used agents include narrow-spectrum beta-lactams for suspected *Staphylococcus aureus* and broader-spectrum coverage for gram-negative organisms (e.g., piperacillin-tazobactam). Vancomycin may be added if methicillin-resistant *Staphylococcus aureus* is suspected. Antibiotic selection is then adjusted based on culture and sensitivity results, and treatment is continued for a total of 4-6 weeks [44]. In refractory or recurrent cases, antibiotic lock therapy may be considered [45].

Surgical Drainage

In addition to antibiotics, surgical drainage of the joint is often necessary to remove infected debris and alleviate swelling [46]. The following procedures are commonly performed: (1) Arthrocentesis: Initial aspiration of joint fluid in all cases to obtain samples and provide temporary relief; (2) Arthroscopy: Utilizes small incisions and a camera to irrigate the joint and perform synovectomy, which allows for faster recovery; (3) Open arthrotomy: In more severe cases, a larger incision is made to perform extensive joint lavage and debridement, this procedure required for more severe cases.

Joint Immobilization

After surgical drainage, the affected knee joint is immobilized using a cast or splint to rest and protect the joint during treatment [47]. Once clinical signs of inflammation improve with antibiotics, knee range of motion exercises are initiated.

Complications, prevention, and prognosis

Complications of septic arthritis include knee, elbow, and hip, subluxation, osteomyelitis, and pathologic fractures [47]. Early diagnosis, prompt administration of antibiotics, and adequate surgical drainage are crucial in minimizing complications and optimizing long-term outcomes [32]. Antibiotic prophylaxis is recommended for high-risk groups and for joint injections [48].

Follow-up

Once children with septic arthritis of the knee have been stabilized through appropriate antibiotic therapy and surgical drainage, close follow-up is necessary to monitor for treatment complications, supervise guide physical therapy, and ensure optimal long-term outcomes.

During Treatment

Initially, patients are typically hospitalized until they become afebrile, experience improved pain and inflammation, and are able to tolerate oral antibiotics. Serial monitoring of white blood cell count, erythrocyte sedimentation rate, and C-reactive protein levels is conducted to assess the response to treatment [44]. If the clinical picture does not improve, follow-up joint aspirates may be performed to assess for persistently elevated synovial fluid markers or resistant organisms [49]. Infections that do not adequately respond to initial antibiotic therapy may require repeat arthrocentesis or surgical washout [38]. Physical therapy is initiated once clinical signs of inflammation subside, aiming to restore range of motion and muscle strength [50].

After Treatment

Follow-up visits are initially scheduled on a weekly to biweekly basis to monitor for complications and assess functional status [51]. Repeat laboratory tests and imaging studies are conducted to rule out residual infection, osteonecrosis, or other sequelae [35]. Physical therapy and rehabilitation continue with a focus on regaining the full range of motion and restoring muscle bulk [46]. Long-term follow-up is necessary to monitor for growth disturbances, arthritis, or recurrent infections that may require orthopedic intervention [52]. Patient education is provided regarding symptoms of potential complications, preventive strategies, and when to seek medical attention again [53].

The prognosis for pediatric septic arthritis depends on factors such as the timing of diagnosis and treatment, the causative organism, and the extent of joint damage. However, with proper long-term follow-up and management, most cases can achieve good functional outcomes.

## Conclusions

Septic arthritis of the knee is a medical emergency in children that necessitates prompt diagnosis and aggressive treatment to minimize joint damage and long-term disability. Despite advancements in managing this condition, it remains challenging for physicians due to nonspecific initial symptoms, variable presentations, and difficulties in diagnosing atypical cases. Reported incidence and prevalence rates likely underestimate the true occurrence of pediatric knee septic arthritis, emphasizing the need for large, well-designed studies to accurately characterize its epidemiology. Standardized diagnostic criteria and improved detection methods may contribute to more precise data in the future.

Timely antibiotic therapy, adequate surgical drainage, and judicious immobilization are the cornerstones of effective treatment. However, achieving a proper balance between these interventions can be challenging, and complications remain common. Early diagnosis and treatment result in better outcomes, underscoring the importance of awareness and a high index of suspicion. Future research should focus on strategies for earlier identification, novel diagnostic biomarkers, immunomodulatory therapies, and other joint-preserving techniques to minimize joint damage and residual disability in children with septic arthritis.

Optimizing long-term follow-up, physical therapy, and patient education can also enhance functional status and quality of life for these patients. Despite the challenges, significant progress has been made in understanding and managing septic arthritis of the knee in children. Continued research efforts, combined with timely diagnosis, appropriate treatment, and close follow-up, offer hope for reducing the burden of this serious condition and improving outcomes for young patients. With a multidisciplinary approach and a focus on innovation, even better results can be achieved in the years to come.
